# Hepatoprotective Potential of Walnut Oil Unsaponifiable Matter on Aging‐Induced Liver Injury via Gut Microbiota–Liver Axis

**DOI:** 10.1002/fsn3.71862

**Published:** 2026-06-05

**Authors:** Junjun Zhou, Dan Hong, Rujin Bai, Xinhui Wang, Shijie Zhu, Qiming Tian, Junxia Xia, Zhou Chen, Siting Li, Yingmin Jia, Aijin Ma

**Affiliations:** ^1^ School of Food and Health Beijing Technology and Business University Beijing China; ^2^ Hebei Key Laboratory of Walnut Nutritional Function and Processing Technology Hengshui China

**Keywords:** aging‐induced liver injury, anti‐aging, gut microbiota, gut‐liver axis, unsaponifiable matter

## Abstract

Aging is characterized by the gradual deterioration of physiological functions. This research aimed to explore the protective effects of unsaponifiable matter (USM), a natural component found in walnut oil, against D‐galactose (D‐gal)‐induced liver injury in aging mice. Male C57BL/6 mice received subcutaneous D‐gal injections alongside oral USM (400 mg/kg) administration for 8 weeks. Systemic evaluations were subsequently conducted using biochemical assays, hepatic transcriptomics, and 16S rRNA sequencing. The results demonstrated that USM administration alleviated hepatic tissue damage, enhanced antioxidant activity, reduced pro‐inflammatory cytokines, and improved serum lipid profiles. Transcriptomic analysis revealed that USM upregulated hepatic energy and lipid metabolism pathways while suppressing inflammatory cascades. Furthermore, USM restored intestinal barrier integrity by upregulating tight junction proteins and rebalanced the gut microbiota, enriching beneficial taxa (e.g., *Akkermansia*, *Lactobacillaceae*) while reducing the relative abundance of *Firmicutes* and *Proteobacteria*. In conclusion, this study elucidates that USM mitigates aging‐associated liver injury by modulating microbial homeostasis and ameliorating intestinal barrier function via the gut‐liver axis.

## Graphical abstract

USM protects against aging‐induced liver injury by modulating the gut‐liver axis. It enriches beneficial microbiota (e.g., *Akkermansia* and *Lactobacillus*) and upregulates tight junction proteins (ZO‐1, Occludin, Claudin) to restore the intestinal barrier. Consequently, this prevents endotoxin influx, effectively reducing hepatic oxidative stress (decreased MDA, increased SOD/GPx) and inflammatory cascades.

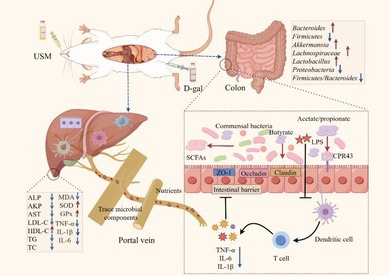

## 1 Introduction

Aging is an inevitable and complex biological process characterized by a gradual decline in physiological functions over time. As the central organ of metabolism, the liver plays a crucial role in providing energy through oxidative metabolism. Meanwhile, it is also vulnerable to oxidative stress, resulting in metabolic disorders and liver injury; therefore, it leads to abnormal liver function (Cheng et al. 2022). The liver and intestine have a shared developmental origin in the preimplantation intestine and are interconnected through the portal venous system, giving rise to the enterohepatic circulation. The maintenance of systemic homeostasis relies heavily on the equilibrium of the enterohepatic axis (Zhang et al. 2022). However, the process of aging disrupts this balance within the gut‐liver axis, resulting in various detrimental effects. These include changes in the composition of intestinal flora, decreased production of short‐chain fatty acids (Sheng et al. 2022), heightened release of inflammatory factors (Ran et al. 2022), impairment of the intestinal barrier (Chen et al. 2022), increased permeability of the intestines, and the tran locaspoation of intestinal microorganisms and their toxic metabolites through the portal vein to the liver (Zou et al. 2023). Ultimately, these alterations contribute to liver injury.

Research has shown that dietary nutrient supplementation can effectively alleviate liver injury caused by aging. For instance, the polysaccharides derived from *Dendrobium hosannae* enhance intestinal metabolite levels and strengthen the intestinal barrier function, while *Angelica sinensis* regulates gut microbiota composition and metabolism. Both interventions improve metabolic health through the gut‐liver axis and suppress age‐related liver injury (Lu et al. 2023; Ma et al. 2023). Consequently, there is a significant interest in investigating dietary interventions aimed at modulating the interactions between the gut and liver. Walnuts are a traditional health food known for their richness in oil. Walnut oil contains unsaponifiable matter (USM), with the USM primarily consisting of phytosterols, tocopherols, squalene, and polyphenols (Zhou et al. 2025). These compounds have garnered significant attention due to their potent antioxidant and anti‐inflammatory properties. Previous studies have demonstrated that unsaponifiable matter derived from wheat bran can reduce lipid accumulation in hepatocytes through the AMPK pathway (An et al. 2023). Additionally, research has shown that γ‐glutamine and unsaponifiable matter from rice bran oil can enhance antioxidant enzyme activities in the liver and colon, while also inhibiting the development of colon cancer in rats (Hung et al. 2012).

In this study, we systematically investigated the effects of walnut oil USM on the gut microbiota and host metabolism, evaluating its protective role against liver injury through the gut‐liver axis in aging mice.

## 2 Methods and Materials

### 2.1 Materials

D‐galactose was purchased from Shanghai Aladdin Biochemical Technology Co. Ltd. in China. Walnut oil was purchased from Hebei Lvling Kangwei Food Co. Ltd. (Hebei, China). Anti‐ZO1 tight junction protein antibody (ab221547), anti‐Claudin 1 antibody (ab307692), and anti‐Occludin antibody (ab216327) were purchased from Abcam (Shanghai, China) Trading Co. Ltd.

### 2.2 Extraction of USM

The extraction of USM was conducted according to the method reported by Zhou et al. (2025). Walnut oil (5 g) was transferred to a flask, followed by the addition of potassium hydroxide solution (1 mol/L, 50 mL) and zeolite. The mixture was heated to reflux for 1 h. After refluxing, the flask was removed from the heat source, and water (50 mL) was added with continuous stirring. Upon cooling, the saponified mixture was transferred to a separating funnel. The flask and zeolite were washed with *n*‐hexane (50 mL), and the resulting washings were combined with the contents of the separating funnel. The mixture was allowed to stand in the funnel until phase separation occurred. The lower aqueous saponification layer was then transferred to a second separating funnel, and the washing procedure was repeated twice. The combined *n*‐hexane extracts was subsequently washed three additional times with a 10% ethanol aqueous solution. The *n*‐hexane extract was subsequently washed three additional times until a drop of phenolphthalein (10 g/L in 95% ethanol) resulted in no color change (from pink to colorless). Finally, the solvent was removed using a rotary evaporator to yield the USM.

### 2.3 Qualitative and Quantitative Analyses of USM

The qualitative and quantitative analyses of USM were conducted according to the method reported by Zhou et al. (2025). USM (50 mg) was dissolved in n‐hexane (5 mL). After dehydration with anhydrous sodium sulfate, the solution (300 μL) was mixed with an equal volume of BSTFA‐TMCS, and silylation was performed at 60°C for 1 h in a water bath. The solvent was then evaporated under nitrogen, and the residue was re‐dissolved in *n*‐hexane (500 μL). The resulting solution was filtered through a 0.22 μm organic membrane and analyzed by gas chromatography–mass spectrometry (GC–MS; 7890A, Agilent Technologies, USA). Separation was performed using a DB‐35ms capillary column (30 m × 0.25 mm, 0.25 μm film thickness). The oven temperature program was set as follows: initial temperature at 90°C for 5 min, increased to 250°C at a rate of 8°C/min, and held for 5 min. The injector temperature was maintained at 300°C. Helium was used as the carrier gas at a constant flow rate of 1.0 mL/min, with an injection volume of 1 μL and a split ratio of 50:1. The mass spectrometer was operated with an interface temperature of 290°C, an ion source temperature of 230°C, and an MS quadrupole temperature of 150°C. The solvent delay time was 3 min, and the mass scanning range (*m/z*) was 35–550.

### 2.4 Animals and Experimental Design

Six‐ to eight‐week‐old SPF‐grade C57BL/6 male mice (20.0–22.0 g) were purchased from Beijing Huafukang Bio‐technology Co. Ltd. (Beijing, China), and all mice were acclimatized and fed for 7 days before the experiment. In order to maintain uniformity in the average body weight of mice in each group, the mice were randomly assigned into three groups (*n*=6 per group). To establish a systemic aging model rather than relying on natural senescence, the mice were randomized as follows. Control group (daily subcutaneous injection of normal saline alongside oral administration of sterile water); Model group (daily subcutaneous injection of 150 mg/kg D‐galactose to induce systemic aging); and USM group (daily subcutaneous injection of D‐galactose + daily oral administration of 400 mg/kg USM), according to previous research (Zhou et al. 2025). All treatments were administered chronically on a daily basis throughout the entire 8‐week experimental period. After the 8‐week feeding period, mice were euthanized by carbon dioxide asphyxiation. Blood was collected via cardiac puncture and allowed to clot at room temperature for 15 min before centrifugation at 3000 rpm for 15 min to obtain serum. Liver and colon tissues were also harvested for subsequent analysis. All protocols pertaining to the utilization of laboratory animals adhered to the guidelines outlined in the eighth edition of the *Guide for the Care and Use of Laboratory Animals* (ISBN‐10: 0‐309‐15396‐4). All experimental animals and treatment methods were approved by the Animal Experiment Ethics Committee of Beijing Huayuanshidai Technology Co. Ltd. (HYSD2021‐2). The study was conducted in strict adherence to institutional and national guidelines for the care and use of laboratory animals, ensuring that all efforts were made to minimize distress and suffering.

### 2.5 Biochemical Analysis

#### 2.5.1 Determination of Liver Function in Mice

Serum samples were collected from mice for liver function tests, and the serum levels of alanine aminotransferase (ALT), aspartate aminotransferase (AST), alkaline phosphatase (AKP), total cholesterol (TC), triglycerides (TG), low‐density lipoprotein cholesterol (LDL‐C), and high‐density lipoprotein cholesterol (HDL‐C) were measured using kits from Nanjing Jiancheng Bioengineering Institute (Nanjing, China).

#### 2.5.2 Determination of Oxidative Stress in Liver

Liver tissues (5 mg) from the mice were collected, homogenized with RIPA buffer (50 μL), and centrifuged (12,000 rpm, 20 min, 4°C). The resulting supernatant was used to measure the concentration of malondialdehyde (MDA) and the enzyme activities of superoxide dismutase (SOD) and glutathione peroxidase (GPx) using kits from Beyotime Institute of Biotechnology (Beijing, China).

### 2.6 Histopathological Analysis

The liver and colon tissues were immersed in 4.0% paraformaldehyde solution overnight for fixation, dehydrated with gradient ethanol, and paraffin‐embedded. Subsequently, the fixed tissues were cut into 5.0‐μm‐thick sections, stained with hematoxylin and eosin (H&E), and then observed and analyzed using with an optical microscope (Zeiss, Oberkochen, Germany; Axio Vert. A1).

### 2.7 Quantitative Real‐Time PCR Analysis

The isolation and extraction of total RNA from liver tissue and colon tissue were performed using an RNA extraction kit, followed by an assessment of RNA concentration and purity. Subsequently, the RNA was converted to complementary deoxyribonucleic acid (cDNA) using the FastKing RT Kit (with DNase). The quantification of mRNA levels was conducted using the SuperReal PreMix Plus (SYBR Green) qPCR kit, and real‐time detection was achieved using the CFX96TM real‐time system with the primer sequences shown in Table S1. The expression level of the target gene was relatively quantified by the 2^−ΔΔCt^ method employing the housekeeping gene glyceraldehyde 3‐phosphate dehydrogenase (GAPDH) as a normalized control, and all experiments were repeated three times.

### 2.8 Western Blotting Analysis

Liver and colon tissues were obtained from mice and subjected to homogenization using tissue lysis buffer, centrifuged (12,000 rpm, 15 min, 4°C) and yielding the supernatant. Equivalent quantities of protein samples were separated by SDS‐PAGE and transferred onto polyvinylidene difluoride (PVDF) membranes through electrophoresis. The PVDF membranes were blocked with 5.0% skimmed milk (room temperature, 2 h). Subsequently, the membranes were subjected to incubation with primary antibodies (4°C, 12 h), followed by incubation with horseradish peroxidase (HRP) conjugated secondary antibodies (room temperature, 2 h). The resulting signals were detected by employing chemiluminescent reagents and visualized with the ImageQuant LAS 500 imaging system (GE Healthcare, USA). To quantify the expression of proteins, the bands on the membranes were subjected to densitometric analysis using ImageJ software.

### 2.9 Transcriptomic Analysis of Liver Tissue

Liver tissues were subjected to extraction using an RNA extraction kit to obtain total RNA. Subsequently, RNA quality was detected and a cDNA library was constructed. The constructed library was then subjected to characterization and sequencing using the Illumina NovaSeq6000 platform (Sinochem, Shanghai, China). The resulting data generated from the Illumina platform were analyzed bioinformatically. To analyze the gene expression levels, the clean reads obtained were compared with the reference genome using HISAT2 software. The FPKM values for gene expression in each sample were computed using featureCounts software for statistical analysis. Intergroup gene differential expression analysis was performed using DESeq2. GO and KEGG enrichment analyses were conducted on the differentially expressed genes (DEGs), employing preset screening thresholds of ∣log2FC∣ > 1 and *p*_adj_ < 0.05.

### 2.10 Analysis of Changes in Intestinal Flora by 16S rRNA Amplicon Sequencing

Microbial genomic DNA was extracted from mouse colon contents, and the V3 ‐ V4 region of the 16S rRNA gene was amplified. Subsequently, sequencing libraries were generated by purifying, quantifying, and homogenizing the PCR amplification products utilizing the Illumina NovaSeq 6000 platform (Illumina, San Diego, CA, USA). The obtained data underwent bioinformatics analysis using BMKCloud (www.biocloud.net) to annotate species, assess abundance through splicing and filtering reads, and clustering or denoising the data to reveal the sample composition.

### 2.11 Statistical Analysis

All data are expressed as mean ± SEM and significant differences between means were analyzed post hoc using SPSS statistical test for multiple comparison tests. Significant differences were indicated by different markers and graphs were plotted using Origin Pro 9.0 software.

## 3 Results

### 3.1 The Composition of USM

**FIGURE 1:**
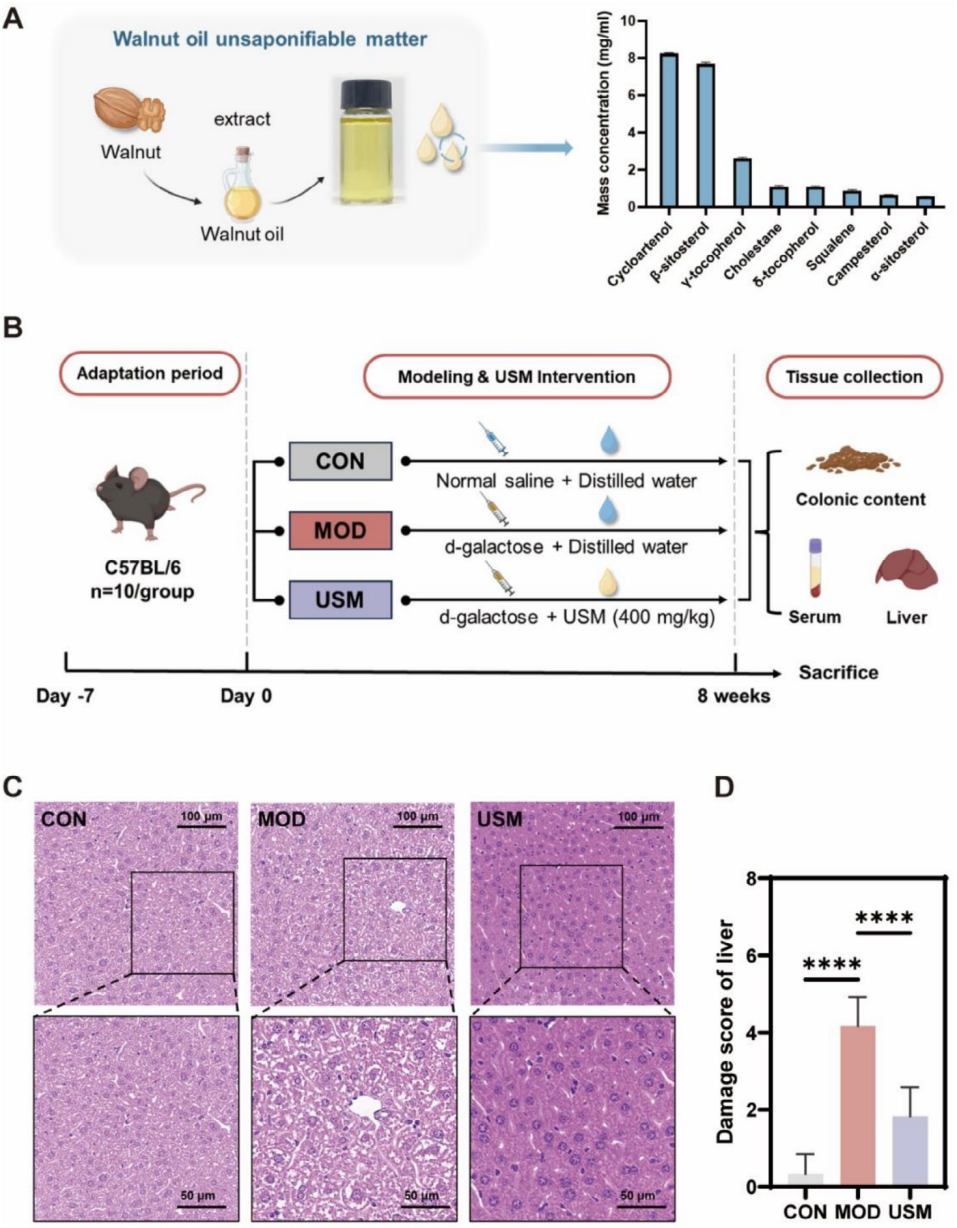
USM alleviated aging‐induced liver injury in mice. (A) Composition and content of USM. USM extract is shown. (B) Schematic diagram of the aging mouse liver injury model. (C) Typical pictures of H&E‐stained liver of each group (Scale bar, 100 and 50 μm). (D) Damage score of histopathology.

The extraction yield of USM from walnut oil was 4.6%. The composition of the USM was analyzed using gas chromatography–mass spectrometry (GC–MS), and the mass concentration of each component was calculated using the peak area normalization method. Based on the mass spectrometry data, the types and concentrations of the compounds in the USM were identified through database matching, as shown in Figure 1A. The results indicate that sterols are the primary components of USM. Cycloartenol, with a mass concentration of 8.25 mg/mL, was the most abundant compound, while *β*‐sitosterol, at 7.69 mg/mL, was the second most abundant compound.

### 3.2 USM Alleviated Liver Injury in Aging Mice

The USM was gavaged to C57BL/6J mice for 8 weeks to prevent aging‐induced liver damage. Over the 8‐week experiment, the mice in the three groups gained weight gradually (Figure 1B). The morphological and structural characteristics of liver tissue undergowent alterations with advancing age. Numerous studies have confirmed the association between liver injury and aging, particularly the gradual decline in liver function manifested as hepatocyte damage, exacerbated inflammatory responses, and impaired metabolic capacity. To investigate these changes, liver tissue was subjected to H&E staining, revealing that the control group exhibited a well‐defined and organized structure characterized by compact hepatocyte arrangement without ballooning degeneration or inflammatory cell infiltration (Figure 1C). The damage scores indicated that the model group had the highest scores, and the damage scores reduced significantly in the USM group (Figure 1D).

### 3.3 USM Ameliorated Hepatic Metabolic Dysfunction in Aging Mice

**FIGURE 2:**
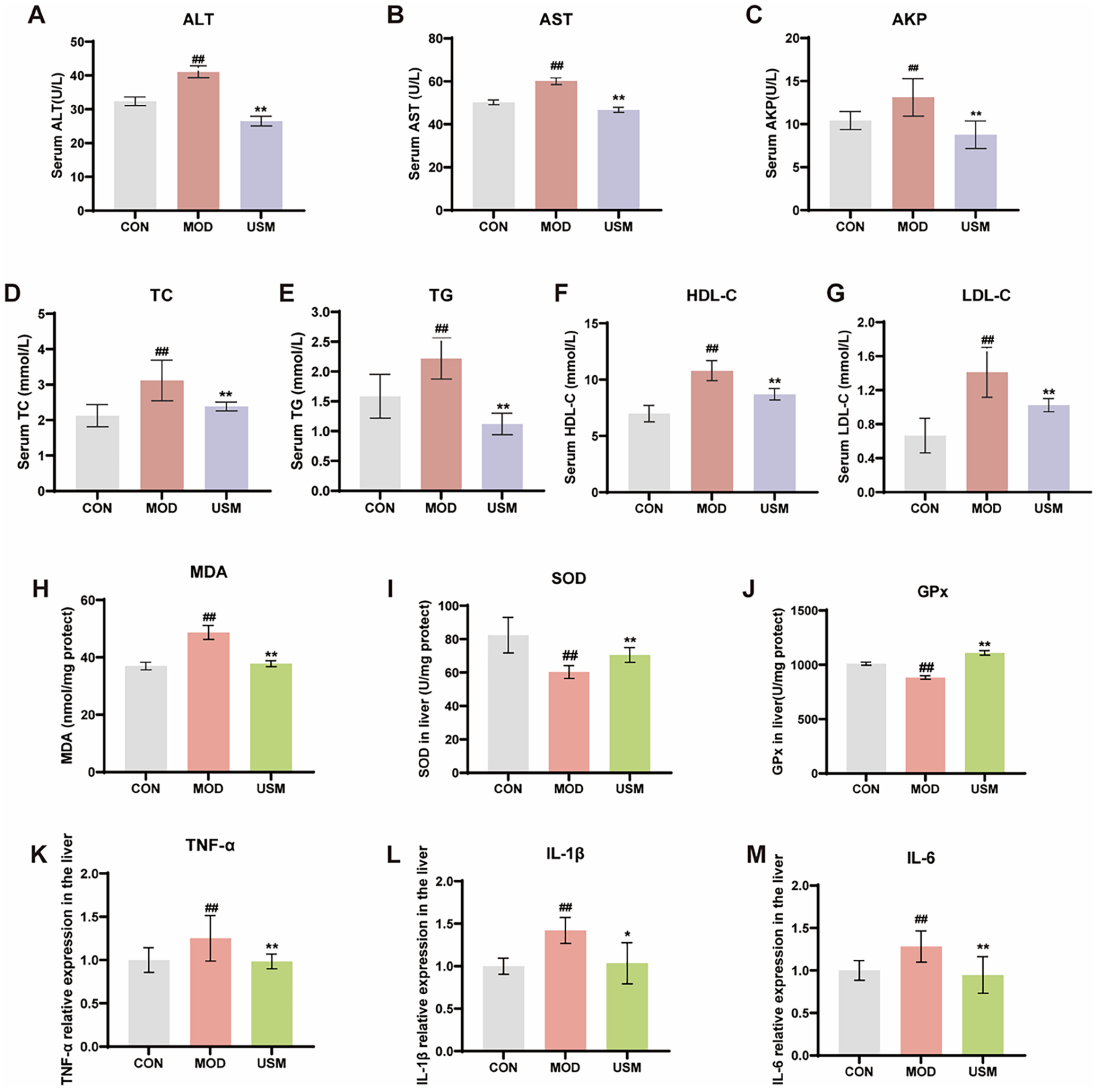
Effects of USM on biochemical indicators in aging mice. (A) ALT in the serum. (B) AST in the serum. (C) AKP in the serum. (D) TC in the serum. (E) TG in the serum. (F) HDL‐C in the serum. (G) LDL‐C in the serum. (H) MDA concentration in the liver. (I) SOD activities in the liver. (J) GPx activities in the liver. (K) TNF‐α mRNA expression level in the liver. (L) IL‐1β mRNA expression level in the liver. (M) IL‐6 mRNA expression level in the liver. Data are presented as mean ± SEM, *n* = 6/group. #*p* < 0.05, ##*p* < 0.01, versus the control group, *p < 0.05, **p < 0.01, versus the model group.

To further investigate the impact of USM on liver function injury in senescent mice, we analyzed serum biochemical markers. The results demonstrated that D‐galactose treatment significantly elevated serum levels of ALT, AKP, and AST compared with the control group (Figure 2A–C), indicating hepatocyte membrane damage and impaired liver function. Notably, USM administration effectively reduced these enzyme levels, suggesting its hepatoprotective role in mitigating cellular damage.

Furthermore, we evaluated lipid metabolism by measuring TC, TG, HDL‐C, and LDL‐C. The D‐gal‐treated group exhibited elevated TC, TG, and LDL‐C levels alongside reduced HDL‐C (Figure 2D–G), reflecting dysregulated lipid homeostasis—a hallmark of aging‐related metabolic dysfunction. USM intervention reversed these abnormalities, restoring lipid balance.

### 3.4 USM Enhanced Antioxidant Capacity and Reduced Inflammation in the Livers of Aging Mice

The experimental results highlight the critical role of oxidative stress in age‐related liver injury (Figure 2H–J). In the aging mouse model, the liver concentration of MDA, a terminal product of lipid peroxidation, increased by 31.53% compared to the control group (Figure 2H). Concurrently, the activities of SOD and glutathione peroxidase GPx decreased by 26.80% and 12.50% compared to the control group, respectively (Figure 2I,J). The elevated MDA levels directly reflect oxidative damage to cellular membranes and organelles, while the decline in SOD (a primary ROS scavenger) and GPx (critical for reducing lipid hydroperoxides) underscores a compromised antioxidant capacity. Notably, USM intervention significantly mitigated these effects. USM reduced liver MDA levels by 22.30% and restored SOD and GPx activities by 16.86% and 25.59%, respectively.

Oxidative stress and inflammation are tightly interconnected in aging‐related pathologies. The excessive ROS production observed in the model group can activate redox‐sensitive transcription factors, which drive the expression of pro‐inflammatory cytokines. As shown in Figure 2K–M, liver levels of TNF‐α, IL‐1β, and IL‐6 increased by 25.10%, 42.10%, and 28.20%, respectively, in aging mice. USM administration effectively suppressed this inflammatory cascade, reducing TNF‐α, IL‐1β, and IL‐6 levels by 21.41%, 27.08%, and 26.03%, respectively.

### 3.5 USM Affected Liver Transcriptome in an Aging‐Liver‐Injured Animal Model

#### 3.5.1 Differentially Expressed Genes Analysis

**FIGURE 3:**
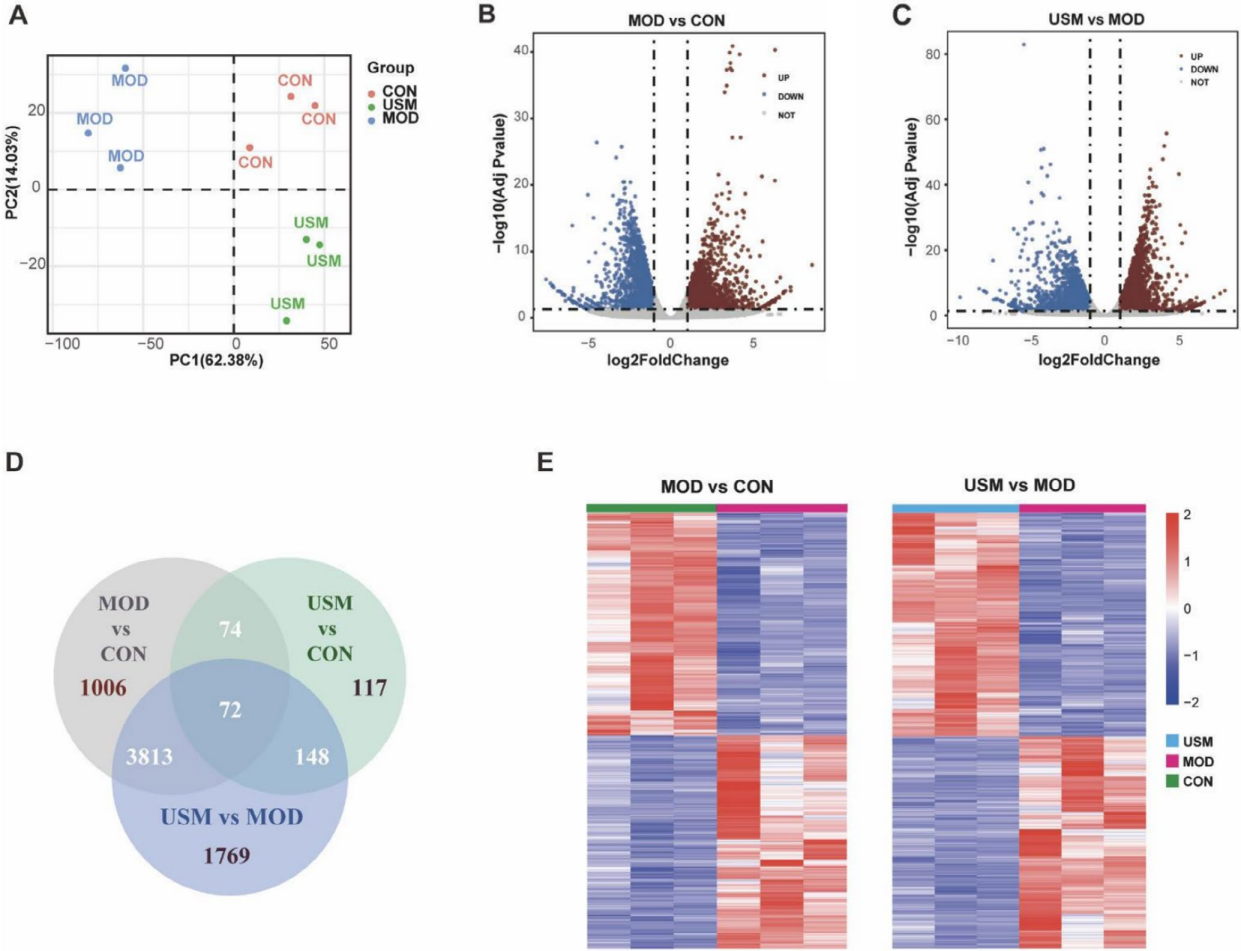
USM affected the DEGs of liver transcriptome. (A) PCA. (B) Model versus Control volcano plot. (C) USM versus Model volcano plot. (D) Venn diagram. (E) Model versus Control and USM versus Model heat map.

To examine the alterations in gene expression during the amelioration of liver injury in aging mice with USM, RNA‐seq was employed to analyze liver tissues. High‐quality sequencing data were obtained (Table S2). Principal component analysis (PCA) and sample similarity analysis revealed distinct segregation among the control, model, and USM groups, indicating significant metabolic disparities between these groups (Figure 3A). Subsequently, differential gene expression analysis was conducted using DESeq2, resulting in the identification of 28,837 genes. The criteria for identifying differentially expressed genes (DEGs) were determined as *p*_adj_ < 0.05 and |log2(foldchange)| > 1. In the control group compared to the model group, a total of 4965 DEGs (2429 up‐regulated and 2536 down‐regulated) were identified. Similarly, when comparing the USM group to the model group, a total of 5801 DEGs (2979 up‐regulated DEGs and 2823 down‐regulated DEGs) were identified. The distribution of these DEGs was visually represented through a volcano polt and heat map (Figure 3B,C,E), while the Venn diagram provided a graphical representation of the DEG distribution in each group (Figure 3D).

#### 3.5.2 GO Pathway Enrichment Analysis of Differentially Expressed Genes

**FIGURE 4:**
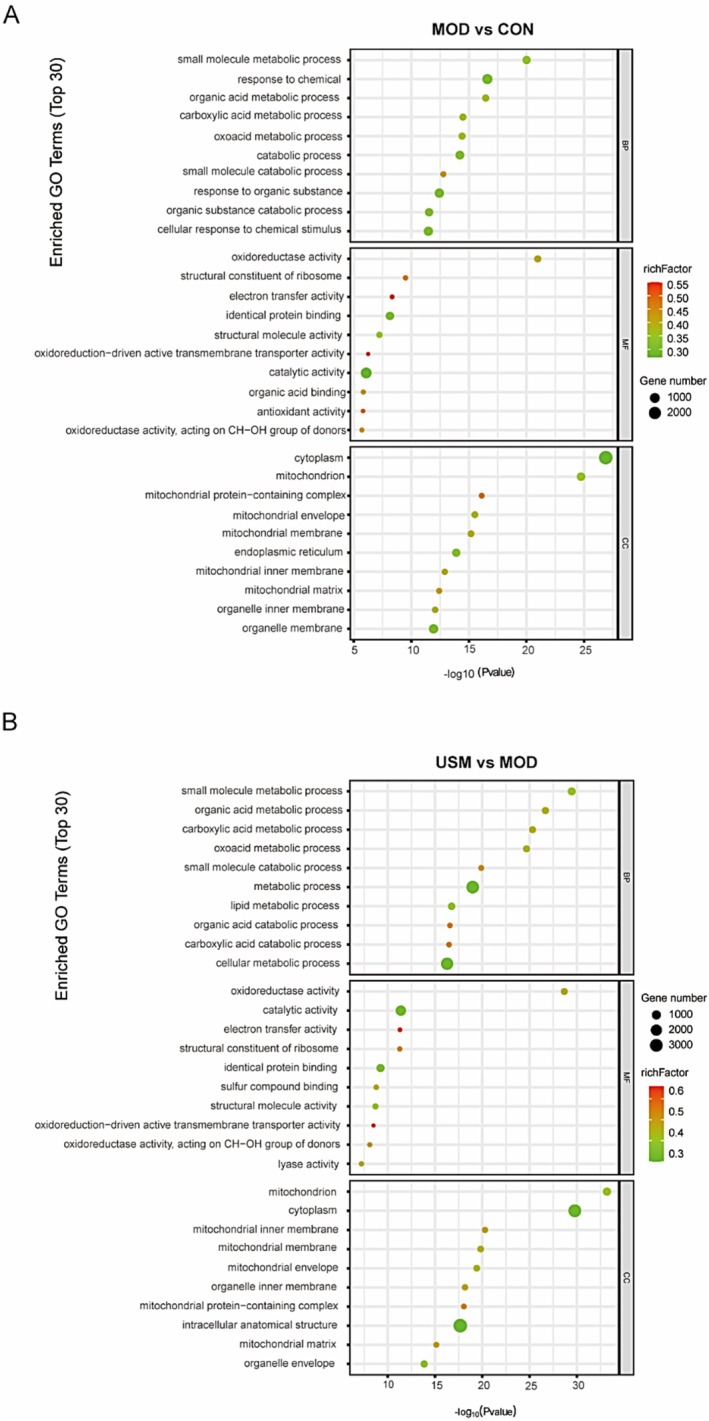
USM affected the GO and KEGG of liver transcriptome. (A) Model versus Control GO enrichment analysis. (B) USM vs. Model GO enrichment analysis.

To gain a deeper understanding of the contributions made by DEGs to biological functions, we conducted functional annotation and enrichment analysis on the DEGs. Specifically, we focused on the top 10 functional entries within the categories of the molecular function (MF), biological process (BP), and cellular components (CC) categories. The functional entries were primarily enriched in carboxylic acid metabolic process, oxoacid metabolic process, lipid metabolic process, organic acid catabolic process, oxidoreductase activity, electron transfer activity, oxidoreduction−driven active transmembrane transporter activity, lyase activity, antioxidant activity, mitochondrion (Figure 4A,B).

#### 3.5.3 KEGG Pathway Enrichment Analysis of DEGs

**FIGURE 5:**
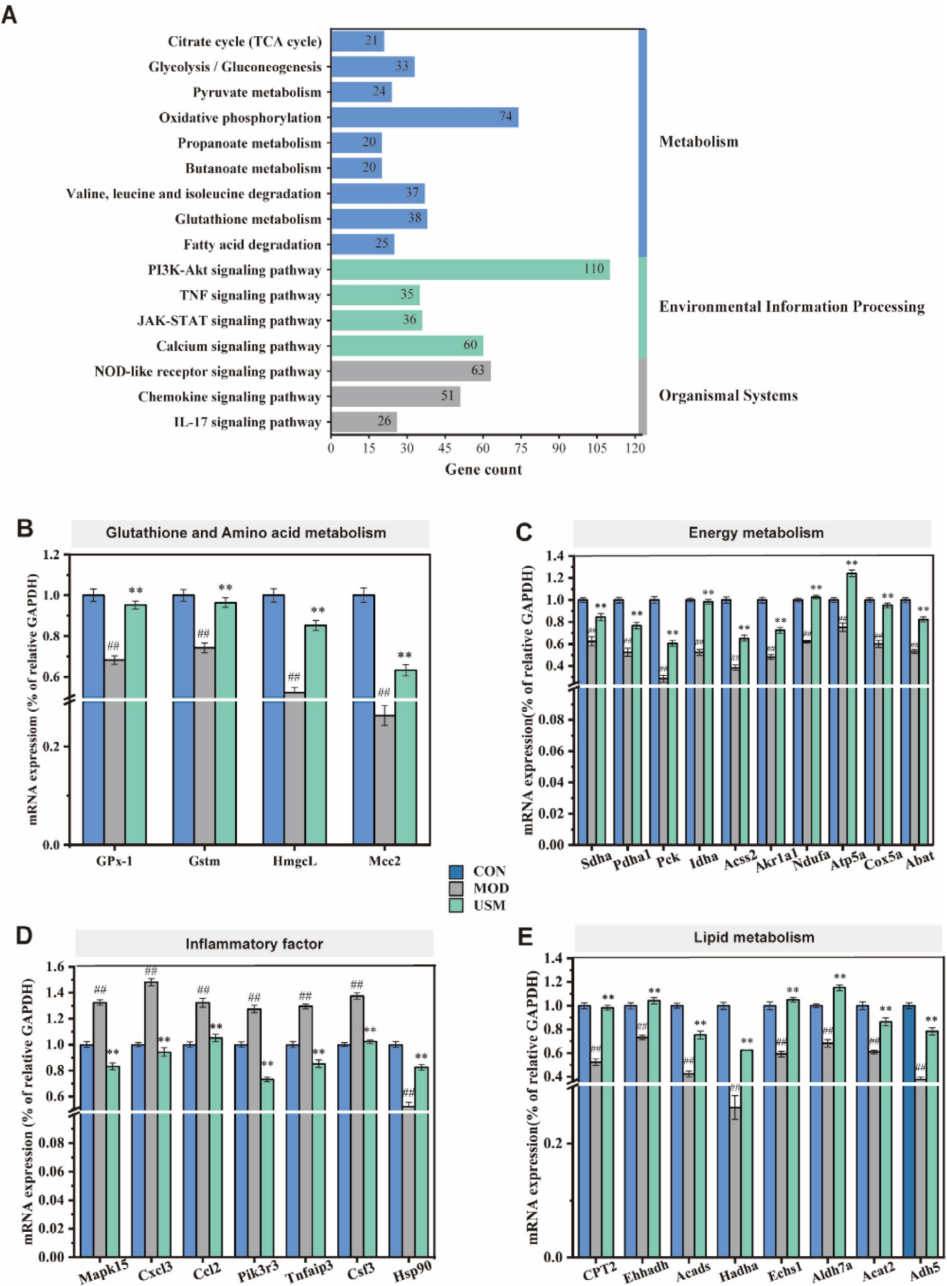
Transcriptomic analysis of liver. (A) The number of genes in each pathway. (B) mRNA expression levels of Glutathione and amino acid metabolism‐related genes. (C) mRNA expression levels of energy metabolism‐related genes. (D) mRNA expression levels of inflammatory factor‐related genes. (E) mRNA expression levels of lipid metabolism‐related genes.

Based on the existing DEGs, KEGG pathways were enriched in MOD vs. CON and USM vs. MOD (up and down TOP 20) (Figure S1), among which KEGG pathways enriched in USM vs. MOD (Figure 5A) contain information on various aspects such as metabolism, genetic information processing, environmental information processing, cellular processes, organismal systems, human diseases, and drug development. The metabolic pathways significantly affected by USM treatment include the citrate cycle (TCA cycle), glycolysis/gluconeogenesis, pyruvate metabolism, oxidative phosphorylation, butanoate metabolism, valine, leucine, and isoleucine degradation, glutathione metabolism, fatty acid degradation, and propanoate metabolism. Additionally, the USM treatment also influences environmental information processing pathways such as the PI3K‐AKT signaling pathway, TNF signaling pathway, JAK–STAT signaling pathway, and Calcium signaling pathway. In organismal systems, the NOD‐like receptor signaling pathway, IL‐17 signaling pathway, and Chemokine signaling pathway were affected. These transcriptomic findings were subsequently validated using qPCR (Figure 5B–E), which confirmed that USM upregulated the expression of genes involved in energy metabolism (*SDHA*, *PDHA1*, *PCK*, *LDHA*, *ACSS2*, *AKR1A1*, *NDUFA*, *ATP5A*, *COX5A*, *ABAT*), amino acid metabolism (*GPx*, *GSTM*, *HMGCL*, *MCCC2*, *NAT8F2*), and lipid metabolism (*CPT2*, *EHHADH*, *ACADS*, *HADHA*, *ECHS1*, *ALDH7A*, *ACAT2*, *ADH5*). Conversely, USM downregulated the expression of genes associated with inflammatory signal transduction and organismal systems, including *MAPK15*, *CXCL3*, *CCL2*, *PIK3R3*, *TNFAIP3*, and *CSF3*, while upregulating *HSP90*.

### 3.6 Effect of USM on Intestinal Tract of Aging Mice

**FIGURE 6:**
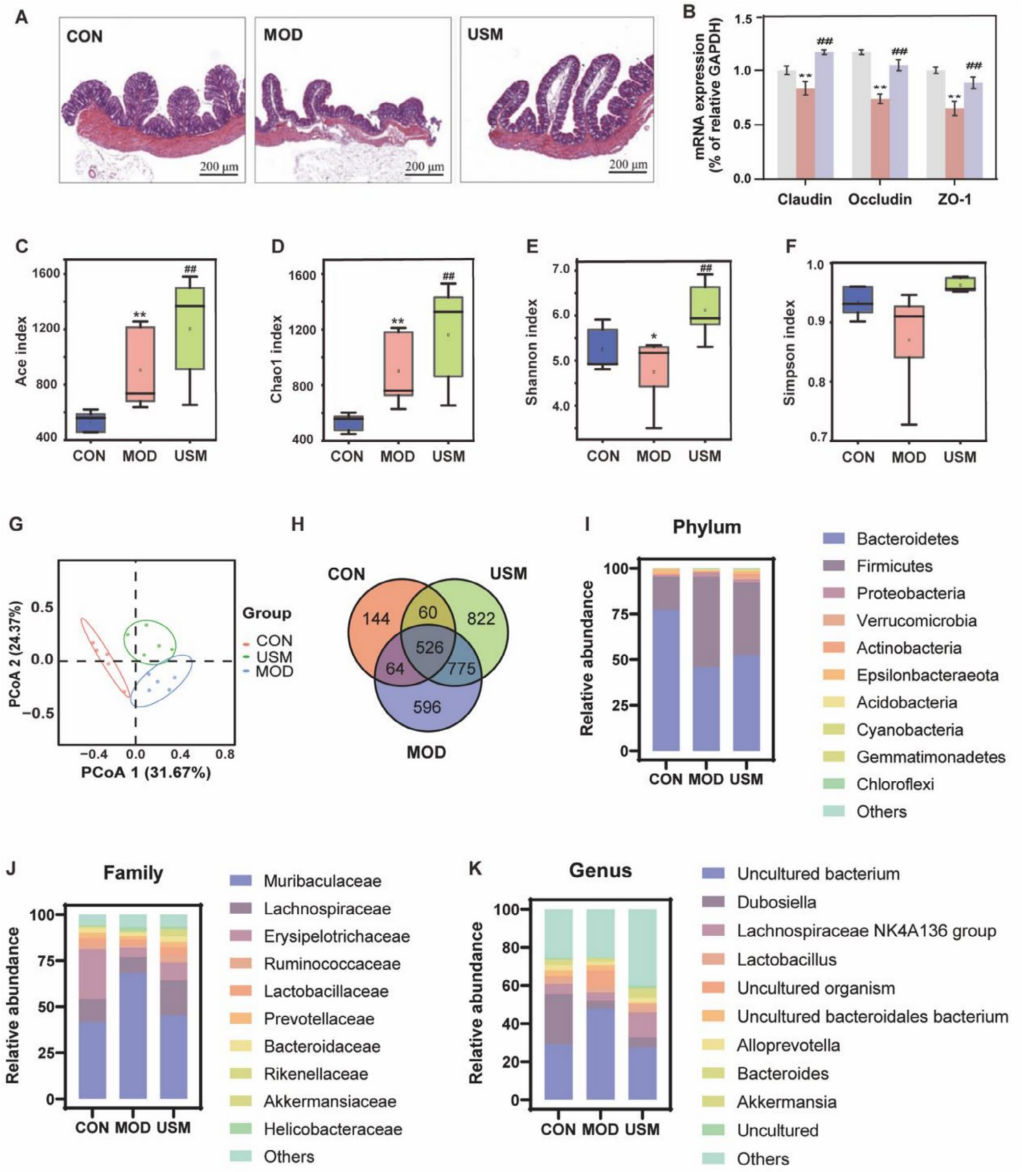
USM alleviates aging‐induced colon damage and dramatically altered gut microbiota composition in mice with aging‐liver injury. (A) H&E staining of colonic tissues. (B) ZO‐1, Occludin and Claudin‐1 mRNA expression levels. (C) Ace index; (D) Chaol index; (E) Shannon index; (F) Simpson index; (G) Analysis for β diversity using the PCoA method; (H) Venn Graph (I) Change in the relative abundance of microbiota at the *phylum* level; (J) Change in the relative abundance of microbiota at the *family* level; (K) Change in the relative abundance of microbiota at the *genus* level; Data are presented as mean ± SEM, *n* = 5/group. *p < 0.05, **p < 0.01, versus the control group, #*p* < 0.05, ##*p* < 0.01, versus the model group.

To investigate the impact of USM on the colonic tissues of aging mice, the results of H&E staining, as depicted in Figure 6A, indicated that the control group exhibited a smooth colon wall without any discernible pathological change alterations in the plasma membrane layer or smooth muscle layer. The glands were orderly arranged, demonstrating regular morphology and distinct structure, with no signs of inflammatory cell infiltration, erosion, or ulceration. Conversely, the model group displayed damage to the intestinal wall of the colon, including partial defects in the plasma membrane layer and a reduction in the number of glands, which were sparsely and haphazardly arranged. However, the colon tissue of the USM group appeared relatively intact, and the cellular arrangement tended to be more complete. These findings indicated that USM has the potential to mitigate age‐related colon damage.

The integrity of the intestinal barrier is a critical determinant of liver health. In this study, the expression of tight junction (TJ) proteins—Occludin, ZO‐1, and Claudin‐1—was assessed to evaluate intestinal barrier integrity in aging mice. As shown in Figure 6B, mRNA levels of Occludin, ZO‐1, and Claudin‐1 decreased by 25.72%, 35.18%, and 16.39%, respectively, in the aging model group. The decline in Claudin‐1, a key regulator of paracellular ion selectivity, suggests compromised selective permeability, while ZO‐1 (a scaffolding protein) and Occludin (transmembrane protein) reductions indicate structural destabilization of TJ. Notably, USM intervention reversed these effects, increasing Occludin, ZO‐1, and Claudin‐1 mRNA levels by 40.39%, 36.49%, and 39.10%, respectively. These findings provide evidence that USM has the potential to mitigate intestinal barrier dysfunction in mice.

### 3.7 Effect of USM on the Structure and Composition of Intestinal Flora

The influence of USM on the composition of the gut microbiota in mice was analyzed through 16S rRNA high‐throughput sequencing. Analysis of the Shannon curve, as depicted in Figure S2, revealed a flattening trend, indicating that the increase in sequencing volume did not correspond proportionally to the number of operational taxonomic units (OTUs). This observation suggests that the obtained data can be effectively employed for subsequent analysis. The impact of USM on the diversity of the gut microbiota in mice was further investigated through alpha diversity measures, as illustrated in Figure 6C–F. The model group demonstrated a significant increase in the Ace and Chao1 indices compared to the control group, while Shannon index exhibited a significant reduction, and Simpson's index didn't show significant changes. These observations indicate that the aging mice exhibited an abundance and diminished diversity in their intestinal flora. The trend was reversed in the USM group. Furthermore, UniFrac's distance‐based principal coordinate analysis (PCoA) and Venn diagram revealed significant differences in microbiome β‐diversity among the three groups, thus indicating substantial differences in bacterial structure between the groups (Figure 6G,H). To analyze the species and their proportions with higher relative abundance within distinct taxonomic levels in each cluster of mouse intestinal flora, a bar chart was constructed to depict the relative abundance of the top 10 microorganisms ranked by abundance at the *phylum*, *family*, and *genus* levels (Figure 6I–K).

**FIGURE 7:**
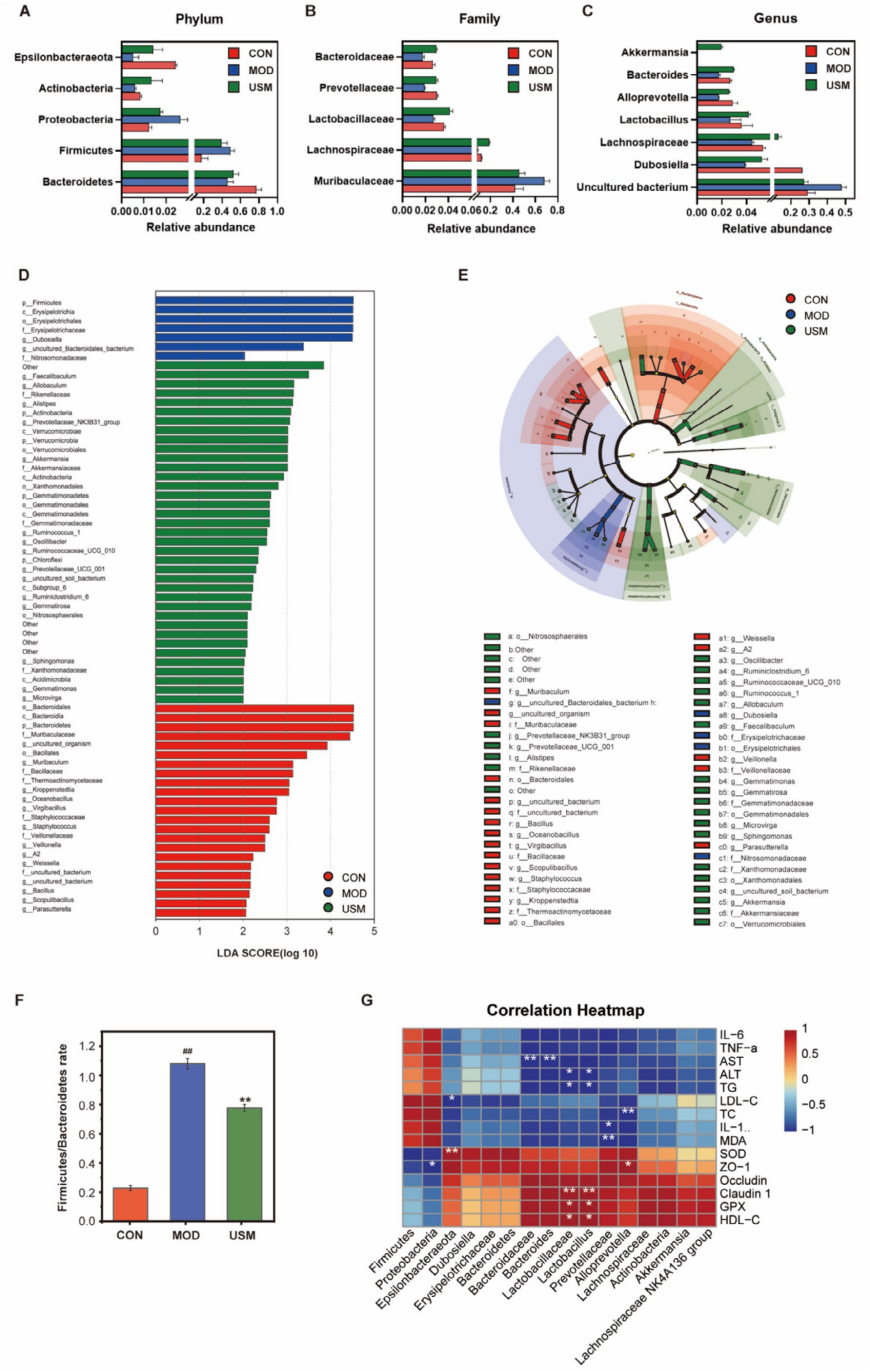
The specific impact of USM on the composition and abundance of microbial communities. (A) The relative abundance of *Firmicutes*, *Bacteroidetes*, *Proteobacteria*, *Actinobacteris* and *Epsilonbacteraeota*. (B) The relative abundance of *Bacteroidaceae*, *Prevotellaceae*, *Lactobacillaceae*, *Lachnospiraceae* and *Muribaculaceae*. (C) The relative abundance of *Akkermansia*, *Bacteroides*, *Alloprevotella*, *Lactobacillus*, *Lachnospiraceae* *NK4A136 group*, *Dubosiella* and *uncultured bacterium*. (D) LDA scores calculated for phylum level features. (E) Evolutionary branching circles. (F) F/B ratio. (G) Correlation analysis between the relative abundance of intestinal microbiota and biochemical criterion.

The results revealed that at the phylum level, the primary dominant phyla across all groups were *Bacteroidetes*, *Firmicutes*, *Proteobacteria*, *Actinobacteria*, and *Epsilonbacteraeota*, collectively constituting more than 96% of the intestinal flora. Compared to the model group, the USM treatment significantly increased the relative abundance of *Bacteroidetes* and *Epsilonbacteraeota* by 14.40% and 170.94%, respectively, while the relative abundance of *Firmicutes Proteobacteria* and the *Firmicutes* to *Bacteroidetes* (F/B) ratio decreased by 19.29%, 34.14% and 29.45% (Figure 7A,F). At the *family* level, the overall dominant flora across all groups consisted of *Muribaculaceae*, *Lachnospiraceae*, *Bacteroidaceae*, *Prevotellaceae*, and *Lactobacillaceae* (Figure 7B). At the *genus* level, the dominant genera in all experimental groups were an *uncultured bacterium*, *Lachnospiraceae NK4A136 group*, *Alloprevotella*, *Bacteroides*, *Akkermansia*, *Dubosiella*, and *Lactobacillus* (Figure 7C).

The impact of USM on the composition of the mouse gut microbiota was examined at the *phylum* and *genus* levels through the utilization of LEfSe analysis (LDA score > 2.0) (Figure 7D,E). A total of 63 taxa were identified as differentially enriched among the three experimental groups. Specifically, at the *phylum* level, the control group exhibited a predominance of *Bacteroidetes*, while *Firmicutes* dominated in the model group. Conversely, the USM group displayed a prevalence of *Actinobacteria*, *Verrucomicrobia*, *Gemmatimonadetes*, and *Chloroflexi*. At the genus level, the control group exhibited dominance of *Muribaculum* and *Kroppenstedtia* bacteria, whereas the model group displayed dominance of *Dubosiella* and *uncultured_Bacteroidales_bacterium*. However, following USM treatment, there was a significant enrichment of *Faecalibaculum*, *Allobaculum*, *Alistipes*, *Prevotellaceae NK3B31 .group*, and *Akkermansia* bacterial groups. These changes in the abundance of intestinal bacterial groups may be closely related to the capacity of USM to ameliorate intestinal barrier dysfunction in mice.

### 3.8 Correlation Analysis Between Intestinal Flora and Biochemical Indexes

To investigate the correlation between USM on the composition of intestinal flora and biochemical markers in aging mice, we analyzed the relative abundance of significantly different microbial species at the *phylum*, *family*, and *genus* levels, as well as the liver levels of oxidative stress indices (GPx, SOD, and MDA), inflammatory factors (IL‐1β, IL‐6, and TNF‐α), liver function‐related indices (AST, ALT, TC, TG, HDL‐C, LDL‐C), and tight junction proteins (ZO‐1, Occludin, and Claudin‐1) in the liver. The results were plotted in a correlation heat map, as shown in Figure 7G. *Epsilonbacteraeota* exhibited a positive correlation with SOD activity, while displaying a negative correlation with LDL‐C. Furthermore, *Bacteroidaceae* and *Bacteroides* demonstrated a negative correlation with AST. Moreover, *Lactobacillaceae* and *Lactobacillus* were both positively correlated with GPx, HDL‐C, and Claudin‐1 and negatively correlated with TG and ALT. Furthermore, *Prevotellaceae* were negatively correlated with MDA and IL‐1β, while *Alloprevotella* were negatively correlated with TC and positively correlated with ZO‐1. Lastly, *Proteobacteria* were negatively correlated with ZO‐1. All the results above collectively prove that the ameliorative action of USM on aging liver injury may be achieved by repair of the gut barrier, regulating the endogenous microflora configurations.

## 4 Discussion

Aging is an inherent and irreversible process of life, accompanied by cessation of the cell cycle, leading to chronic inflammation and organ dysfunction. The liver is one of the vital organs that play a key role in energy metabolism (Zhao et al. 2022). The dysbiosis of intestinal flora caused by aging leads to an imbalance of cellular metabolism and degeneration of liver function. In this study, we investigated the potential mechanism of USM to ameliorate liver injury in aging mice by maintaining the gut‐liver axis homeostasis in terms of oxidative stress, inflammatory factors, and intestinal flora.

ALT and AST, which are important transaminases in the liver, serve as biochemical markers of liver injury. Increasing the serum levels of ALT and AST indicate hepatocyte injury, while high levels of TC, TG, and LDL‐C or low levels of HDL‐C indicate dyslipidemia. In this study, it was observed that in the USM group, serum TC, TG, ALT, AST, and LDL‐C levels were decreased and HDL‐C levels were increased. The above results show that USM may possess a hepatoprotective effect and the ability to modulate lipid metabolism in aging mice. Histopathological analysis of H&E staining was consistent with the previous reports, revealing enlarged interstitial spaces between hepatocytes and severe inflammatory cell infiltration in the model group (Zhu, Hong, et al. 2022). These pathological changes were alleviated in the USM group, indicating that USM has a protective effect on the liver and alleviated the phenomenon of enlarged interstitial spaces between hepatocytes and the presence of inflammatory cell infiltration. These results confirmed the successful establishment of the aging model and provide evidence for the protective effect of USM against senescence‐induced liver injury.

Liver injury elicits a liver inflammatory response, where the levels of pro‐inflammatory cytokines IL‐1β, IL‐6, and TNF‐α serve as direct indicators of the extent of inflammation. Previous investigations have demonstrated that swainsonine induces liver inflammation and upregulates the expression levels of TNF‐α and IL‐1β in the liver. In the current investigation, the concentrations of pro‐inflammatory factors IL‐1β, IL‐6, and TNF‐α were quantified, and it was observed that USM treatment significantly reduced their release from liver tissues, thereby mitigating hepatocyte inflammation. The lipid peroxidation product MDA serves as an indirect indicator of oxidative harm and is influenced by the body's antioxidant capacity. The excessive accumulation of MDA has the potential to disrupt cellular membrane integrity and induce cellular damage (Li, Zhou, et al. 2019). Essential components of the antioxidant defense system, such as SOD, GPx, and CAT, actively safeguard cells and tissues against oxidative injury. Our study aligns with previous investigations, which have reported an elevation in MDA concentration and a decline in SOD and GPx enzyme activities during oxidative stress (Tewari et al. 2020; Zakaria et al. 2021). In the present study, the administration of USM treatment resulted in a decrease in the concentration of MDA and an increase in the activity of SOD and GPx enzymes within the model group. This intervention effectively alleviated oxidative damage in the liver. This suggests that USM exerts dual antioxidant effects: (1) scavenging ROS to reduce lipid peroxidation, and (2) enhancing the activity or expression of antioxidant enzymes. Such effects may be attributed to bioactive components in USM, such as vitamin E (a lipid‐soluble antioxidant) and squalene (a cholesterol precursor with ROS‐neutralizing properties) (Niki 2014). These compounds likely activate related signaling pathway, a master regulator of antioxidant gene expression, thereby upregulating SOD and GPx (Wu et al. 2022).

Multiple studies have demonstrated the significant involvement of host genes in the regulation of disease pathogenesis in organisms (Li, Li, et al. 2019). Hepatic transcriptomic analysis of differentially expressed genes (DEGs) revealed that USM upregulated several metabolic pathways (including Citrate cycle (TCA cycle), Glycolysis/Gluconeogenesis, Pyruvate metabolism, Oxidative phosphorylation, Butanoate metabolism, Valine, leucine and isoleucine degradation, Glutathione metabolism, Fatty acid degradation, and Propanoate metabolism), while downregulating multiple inflammatory pathways (PI3K‐AKT, TNF, JAK–STAT, NOD‐like receptor, IL‐17, and Chemokine signaling pathways), which is consistent with the biochemical findings. Prior investigations have demonstrated that the G‐protein‐coupled receptor GPRC6A exerts direct control over glucose and metabolic pathways within the liver, alongside the synthesis and secretion of FGF‐21, thereby contributing to the maintenance of energy homeostasis (Wang, Lan, et al. 2023). Moreover, it has been discovered that Ginseng Baijusan (SLBZP) exerts its regulatory effects on liver mitochondrial energy metabolism in mice with non‐alcoholic fatty liver disease (NAFLD) through the UCP2/AMPK/IF1 signaling pathway. This mechanism enhances liver mitochondrial membrane permeability, respiratory function, and oxidative phosphorylation efficiency (Yao et al. 2023). The impairment of metabolism regulation due to liver cell damage results in disturbances in liver energy metabolism, subsequently leading to the onset of liver inflammation and oxidative stress (Zhang et al. 2021). USM appears to counteract this by transcriptionally reprogramming hepatic metabolism.

The gut‐liver axis functions as a channel for reciprocal communication between the intestinal tract and the liver, thereby preserving the equilibrium of the gut microbiota and ensuring the stability of liver metabolic processes. Disruption of the delicate equilibrium of intestinal flora can lead to detrimental consequences, including impairment of the host's intestinal barrier and disturbance of metabolic processes. The pivotal function of the gut barrier lies in its ability to regulate host metabolism by preventing the escape of detrimental entities, such as microorganisms and metabolites, from the intestinal tract (Liu et al. 2022). The maintenance of the integrity of the intestinal barrier and the permeability of cells is attributed to the presence of tight junction proteins, such as ZO‐1, claudin‐1, and occludin, between intestinal epithelial cells. Several studies have demonstrated that the expression of tight junction proteins can be diminished by D‐galactose, leading to the disruption of their functionality and an increase in intestinal permeability. Consequently, this can facilitate the translocation of lipopolysaccharides (LPS) from the intestine to the liver through the portal circulation, ultimately eliciting an inflammatory response in the liver (Sun et al. 2023). Several functional food ingredients have demonstrated the ability to enhance the integrity of the intestinal barrier. For example, sesquiterpenes have been documented to inhibit the initiation of endotoxin‐mediated activation of the Toll‐like receptor‐associated IKKα/NF‐κB p65 pathway, thereby, mitigating intestinal barrier dysfunction and promoting favorable alterations in the composition of the intestinal microbiota (Yang et al. 2023). Another study revealed that the water‐soluble extract derived from the leaves of the soft radish ( *Corchorus olitorius* L.) (WM) alleviated symptoms of alcohol‐induced colitis in mice. In addition to mitigating colonic inflammatory response and oxidative stress, it also safeguarded the integrity of the intestinal barrier (Do et al. 2021). Within our investigation, the utilization of USM stimulated the mRNA upregulation of tight junction proteins (Claudin‐1, Occludin, and ZO‐1), resulting in decreased intestinal permeability, enhanced intestinal barrier functionality, restoration of impaired intestinal ultrastructure, and preservation of normal colonic tissue function.

The gut microbiota is a complex ecological system comprosed of bacteria, archaea, eukaryotes, and viruses. Notably, *Bacteroidetes, Firmicutes, Actinobacteria*, and *Proteobacteria* collectively constitute 98% of the overall microorganism population (Zhu, Thakur, et al. 2022). These microorganisms inhabit the host's intestinal tract and exert significant influence on the host's nutrient metabolism (Wang, Lan, et al. 2023). Perturbation of the gut flora can disrupt the intestinal barrier function, thereby predisposing the host to liver injury (Do et al. 2021). Our research findings indicate a notable increase relative to the abundance of *Bacteroidetes*, *Actinobacteria*, *Epsilonbacteraeota*, *Lachnospiraceae*, *Erysipelotrichaceae*, *Ruminococcaceae*, *Lactobacillaceae*, *Prevotellaceae*, *Bacteroidaceae*, *Dubosiella*, and *Akkermansia*, while the relative abundance of *Firmicutes* and *Proteobacteria* exhibited a decrease. *Bacteroidetes* and *Firmicutes* are recognized as two of the most prevalent bacterial taxa in the gut microbiota (Qi et al. 2022). *Bacteroides* is a beneficial bacterium that plays a significant role in the production of short‐chain fatty acids, particularly acetate and propionate, within the gastrointestinal tract (Xia et al. 2022). These short‐chain fatty acids contribute to the maintenance of homeostasis in the gut. The current study revealed a negative correlation between the relative abundance of *Bacteroidetes* and serum levels of AST, as indicated by Pearson correlation analyses. This finding implies that *Bacteroidetes* play a crucial role in preserving intestinal homeostasis through the mitigation of metabolite translocation from the intestinal flora and safeguarding the liver. A study by Ting Xia et al. (2022) demonstrated that Ginsenoside (GRg1) diminishes inflammation levels in the intestines and alleviates intestinal dysfunction by augmenting the relative abundance of *Bacteroides*. *Firmicutes*, a significant contributor of butyrate, serves as a primary nutrient source for intestinal epithelial cells, thereby upholding cellular barrier integrity, and is abundantly present in a healthy gut. The *Firmicutes* to *Bacteroidetes* (F/B) ratio is commonly employed as a means of evaluating the gut's health status (Zhu, Hong, et al. 2022). A significant alteration in the F/B ratio indicates a dysbiosis of the intestinal flora, which may be accompanied by the onset of various disorders, such as obesity, inflammation, and metabolic disorders (Mandato et al. 2021; Zhang et al. 2022). In the present study, after the intervention of USM, there was an observed increase in the relative abundance of *Bacteroidetes* in the intestines of senescent mice, while both *Firmicutes* and F/B values experienced a significant decrease. Additionally, the level of inflammation was reduced, and USM demonstrated a mitigating effect on aging‐induced intestinal inflammation and metabolic disorders.

*Akkermansia*, a bacterium that enzymatically breaks down mucin and serves as a source of energy for other commensal microorganisms, assumes a pivotal role in the process of healthy aging (Gurwara et al. 2020). This bacterium is responsible for the production of short‐chain fatty acids, preservation of the structural integrity of the intestinal epithelium, and stimulation of epithelial cells to secrete mucus. In the elderly population, a reduction in the relative abundance of *Akkermansia* within the gastrointestinal tract can give rise to a condition known as “leaky gut”, which subsequently leads to elevated levels of systemic inflammation commonly referred to as “inflammatory aging” (Liu et al. 2022). Following intervention by USM, aging mice exhibited a heightened relative abundance of *Akkermansia*, which facilitated the preservation of the intestinal barrier and hindered the interaction between pathogens and the intestinal mucosa, consequently safeguarding the well‐being of the host. Prior investigations have documented that Mangiferin augmented the prevalence of *Akkermansia*, fortified the functionality of the intestinal barrier, diminished the influx of LPS into the circulation, and contributed to the anti‐inflammatory properties of mangiferin (He et al. 2023).

*Lachnospiraceae* and *Lactobacilli* exhibit probiotic properties through the production of butyrate, which can enhance intestinal function, alleviate age‐related enteritis, and provide additional protection to the intestinal barrier (Li et al. 2021; Reuter and Bajaj 2020; Wang, Lan, et al. 2023). The abundance of *Lachnospiraceae* and *Lactobacilli* decreases in aging mice, but intervention with USM leads to a significant increase in both species. Furthermore, there exists a positive correlation between the relative abundance of *Lactobacillus* and the concentrations of GPx, HDL‐C, and Claudin‐1, while a negative correlation is observed with the concentrations of ALT and TG. These findings imply that *Lactobacillus* plays a role in enhancing intestinal barrier function, mitigating the exposure of harmful substances in the intestine to the liver, and maintaining proper liver metabolism. Previous studies have also reported an association between Lactobacillus and the increase in Phytic acid (PA), as well as the reduction of liver steatosis (Okazaki and Katayama 2020; Wang et al. 2020). However, *Proteobacteria*, a pathogenic bacterium, exhibited a substantial rise in abundance within the intestinal microbiota of patients with inflammation (Zhang et al. 2022). In our study, the relative abundance of *Proteobacteria* was reduced in the USM group, displaying a negative correlation with ZO‐1. *Proteobacteria* can disrupt tight junction proteins in the intestines, consequently exacerbating damage to the intestinal barrier.

Notably, rather than simply restoring the microbiota to the control baseline, these collective microbial changes indicate that USM establishes a new microbial homeostasis. This distinct profile represents a compensatory protective adaptation against aging‐induced stress. Consequently, by reshaping this microbial balance, USM mitigates intestinal inflammation, increases the expression of intestinal tight junction proteins, and ameliorates intestinal dysfunction.

Overall, our findings establish a foundation for future research. The identification of USM and its hepatoprotective effects against aging informs targeted nutraceutical development, while its gut microbiota modulation reveals novel intervention strategies. However, limitations persist: (1) Gut microbiota‐host interactions exhibit context‐dependent complexity, necessitating broader investigations—including intestinal barrier function, immunophenotyping, metabolomics, and fecal microbiota transplantation—to elucidate USM's mechanistic antagonism of aging‐related liver injury; (2) Longitudinal assessment of USM's efficacy and safety in aging‐liver models remains imperative to validate sustained benefit.

## 5 Conclusion

**FIGURE 8:**
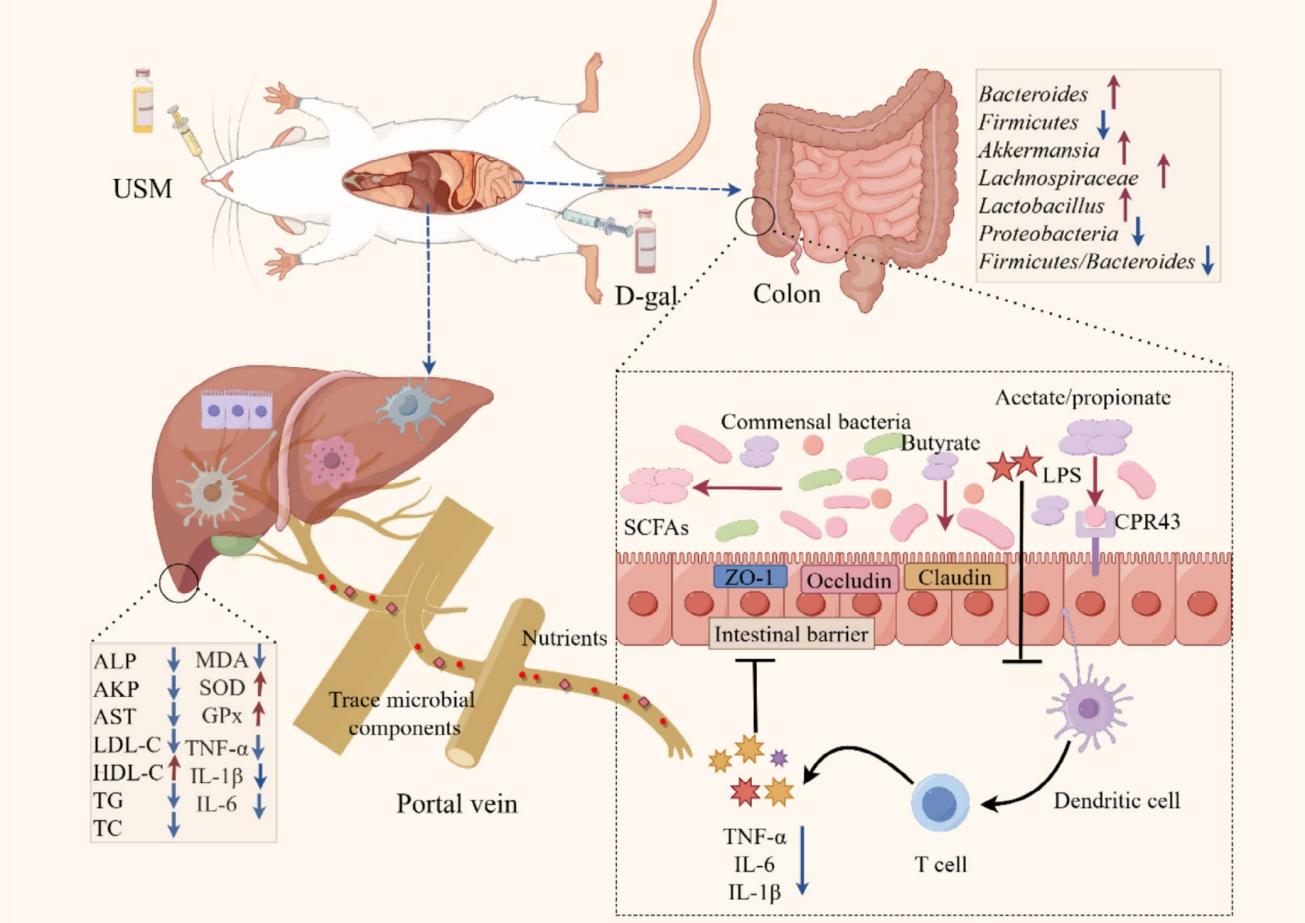
The proposed mechanism of USM to alleviate aging‐injury liver.

In summary, the present study demonstrates that USM alleviates aging‐induced liver injury by synchronously targeting oxidative stress, intestinal barrier dysfunction, and microbial dysbiosis within the gut‐liver axis (Figure 8). Specifically, USM enhances liver antioxidant capacity by upregulating SOD and GPx activities while reducing MDA accumulation, and it effectively decreases pro‐inflammatory cytokines (TNF‐α, IL‐1β, IL‐6) as well as serum lipids (TC, TG, LDL‐C). Concurrently, USM repairs intestinal barrier integrity through the elevated expression of tight junction proteins (ZO‐1, Occludin, Claudin‐1). Furthermore, USM rebalances gut microbiota composition by increasing the relative abundance of beneficial taxa (e.g., *Akkermansia*, *Lactobacillus*, and *Bacteroidetes*) while suppressing pathogenic *Proteobacteria*, collectively normalizing the *Firmicutes*/*Bacteroidetes* ratio. Ultimately, these findings provide a robust scientific basis for considering USM as a potential dietary supplement in the development of anti‐aging functional foods.

## Author Contributions

**Rujin Bai:** investigation, resources. **Xinhui Wang:** methodology, formal analysis. **Yingmin Jia:** funding acquisition. **Aijin Ma:** supervision, writing – review and editing. **Qiming Tian:** conceptualization. **Shijie Zhu:** resources. **Dan Hong:** investigation, resources. **Junxia Xia:** conceptualization. **Junjun Zhou:** writing – original draft, investigation, data curation, formal analysis. **Zhou Chen:** writing – review and editing. **Siting Li:** project administration.

## Funding

This work was supported by National Key Research and Development Program of China, 2022YFD1600402. The Chinese Red Cross Foundation ‘Six Walnuts·National Brain Nutrition Research Fund’.

## Conflicts of Interest

The authors declare no conflicts of interest.

## Supporting information


**Table S1:** Primer sequences used for qRT‐PCR Analysis.
**Table S2:** Raw data table of transcriptomics analysis.
**Figure S1:** Shannon curves for mouse fecal samples.
**Figure S2:** USM affected the GO and KEGG of liver transcriptome. (A) Model versus Control KEGG enrichment analysis—up‐regulation; (B) USM versus Model KEGG enrichment analysis—up‐regulation; (C) Model versus Control KEGG enrichment analysis—down‐regulation; (D) USM versus Model KEGG enrichment analysis—down‐regulation.

## Data Availability

The data that support the findings of this study are available from the corresponding author upon reasonable request.
