# Supplementary material for: Hepatoprotective Potential of Walnut Oil Unsaponifiable Matter on Aging‐Induced Liver Injury via Gut Microbiota–Liver Axis
Source: Food Sci Nutr. 2026 Jun 5;14(6):e71862. doi: 10.1002/fsn3.71862 (PMC13238569; doi:10.1002/fsn3.71862)

**Hepatoprotective potential of walnut oil unsaponifiable matter on aging-induced liver injury via gut microbiota-liver axis**

**Supplementary Table Captions**

**Table.S1 Primer sequences used for qRT-PCR Analysis.**

**Table.S2 Raw data table of transcriptomics analysis.**

**Table. S1 Primer sequences used for qRT-PCR Analysis**

|  | **forward 5′ – 3′** | **reverse 5′ – 3′** |
| --- | --- | --- |
| TNF-a  a | CTCATGCACCACCATCAAGG | ACCTGACCACTCTCCCTTTG |
| IL-1β | AGCTGGAGAGTGTGGATCCC | CCTGTCTTGGCCGAGGACTA |
| Il-6 | CCACTTCACAAGTCGGAGGC | GGAGAGCATTGGAAATTGGGGT |
| ZO-1 | TATTATGGCACATCAGCACG | TGGGCAAACAGACCAAGC |
| Occludin | CTGCAGCTACTGGACTCTAC | GGACTGTCAACTCTTTCCAC |
| Claudin 1 | GACAAC ATCGTGACSGCCCAG | WGCCCAGCCARTGAAGAGRGC |
| Sdha | AGCCTCAAGTTCGGGAAAGG | CACAGTGCAATGACACCAAC |
| Pdha1 | GAGTGAGTTACCGTACCCGAGAA | ACTGGCGAGATTGCTGTTCA |
| Pck | TGCCAGCCAGACTCCATTCACA | ACTCTCCACACGAGCCACATCC |
| Ldha | GCCTGTATGGAGTGGAGTGAATG | GTGAACCGCTTTCCACTGTTC |
| Acss2 | AAAGGAGCAACTACCAACATCTG | GCTGAACTGACACACTTGGAC |
| Akr1a1 | TCTACGGCAATGAGCCT | GTAGGCTACCGCCACATTGATT |
| Ndufa4 | AGTCCGTAGTGTCTCATTG | TACAGTGTTGCTCCAGTAG |
| Atp5a | GTCCGCCTACATTCCAACAA | GACACAGACAAGCCCACATTA |
| Cox5a | GGGTCACACGAGACAGATGA | ACFACCTCCAAGATGCGAAC |
| Abat | GGAGCATCGGAAGGTGATCG | CCCGCGTCCTGATTAGATGG |
| Cpt2 | ATGGCACTATGCACCTACCC | CGGGCACGTTTCTTAGCATC |
| Ehhadh | CCTGGGCTGTCACTATAGGATT | AGAAGCTGGGTTCCTCTTGC |
| Acads | CGGCAGTTACACACCATCTAC | GCAATGGGAAACAACTCCTTCTC |
| Hadha | ATATGCCGCAATTTTACAGGGT | ACCTGCAATAAAGCAGCCTGG |
| Echs1 | GCTGCTGTCAATGGCTATGC | ACCAGTGAGGACCATCTCCA |
| Aldh7a | CGAGCCAATAGCAAGAGTCC | CTTCACCCACACCTTCCACT |
| Acat2 | CCCGTGGTCATCGTCTCAG | GGACAGGGCACCATTGAAGG |
| Adh5 | GTCACACAGATGCCTACACTC | GCCCCGCAACTTTGCAGCCC |
| Mapk15 | TGTTTGAGTCCATGGACACC | GCATCCAATAGAACGTTGGC |
| Cxcl3 | CGCCCAAACCGAAGTCAT | GTGCTCCCCTTGTTCAGTATC |
| Ccl2 | CTTCTGGGCCTGCTGTTCA | CCAGCCTACTCATTGGGATCA |
| Pik3r3 | GAGAGGGGAATGAAAAGGAGA | ATCATGAATCTCACCCAGACG |
| Tnfaip3 | AAACCAATGGTGATGGAAACTG | GTTGTCCCATTCGTCATTCC |
| Csf3 | GCTGCTGCTGTGGCAAAGT | AGCCTGACAGTGACCAGG |
| Hsp90 | AGTCCCAGTTCATTGGCTAC | TCCAGTCATTGGTGAGGCT |
| Gpx-1 | TCTACCTGGTAACTTTCGAGCAA | CCTTTATTGCAGAGCCTCCTT |
| Gstm | AGAACCAGGCCATGGACTTCAG | CCAGCAAACCACTTCCTGTCTC |
| Hmgcl | ATTGTGGAAGTTGGTCCTCGA | GGACCCAGTGGCTCACAGTT |
| Mcc2 | CCAACGAAACAACATTCACG | CACAGATGCGAGGTCGAGTA |
| Gapdh | ACTTTGGTATCGTGGAAGGACT | GTAGAGGCAGGGATGATGTTCT |

**Table.S2 Raw data table of transcriptomics analysis**

| Sample | Raw_reads | Clean_reads | Clean_bases | Error(%) | Q20(%) | Q30(%) | GC(%) |
| --- | --- | --- | --- | --- | --- | --- | --- |
| Control1 | 62273740 | 61620926 | 9.23G | 0.04 | 98.52 | 95.36 | 48.18 |
| Control2 | 63045222 | 62503138 | 9.35G | 0.04 | 98.59 | 95.57 | 48.71 |
| Control3 | 59975792 | 59322858 | 8.88G | 0.04 | 98.56 | 95.53 | 48.18 |
| USM1 | 63247298 | 62679158 | 9.37G | 0.04 | 98.7 | 95.95 | 50.71 |
| USM2 | 51832650 | 51368644 | 7.69G | 0.04 | 98.69 | 95.89 | 50.34 |
| USM3 | 57269970 | 56794010 | 8.48G | 0.04 | 98.82 | 96.35 | 51.59 |
| Model1 | 59799928 | 59214868 | 8.86G | 0.04 | 98.57 | 95.56 | 47.12 |
| Model2 | 51062770 | 50592182 | 7.58G | 0.04 | 98.33 | 94.75 | 43.6 |
| Model3 | 55365316 | 54888756 | 8.21G | 0.04 | 98.47 | 95.23 | 45.95 |

**Supplementary Figure Captions**

**Fig.S1 Shannon curves for mouse faecal samples.**

**Fig.S2 USM affected the GO and KEGG of liver transcriptome.** **(A)** Model vs Control KEGG enrichment analysis - up-regulation; **(B)** USM vs Model KEGG enrichment analysis – up-regulation; **(C)** Model vs Control KEGG enrichment analysis - down-regulation; **(D)** USM vs Model KEGG enrichment analysis - down-regulation.

**Fig.S1 Shannon curves for mouse faecal samples.**


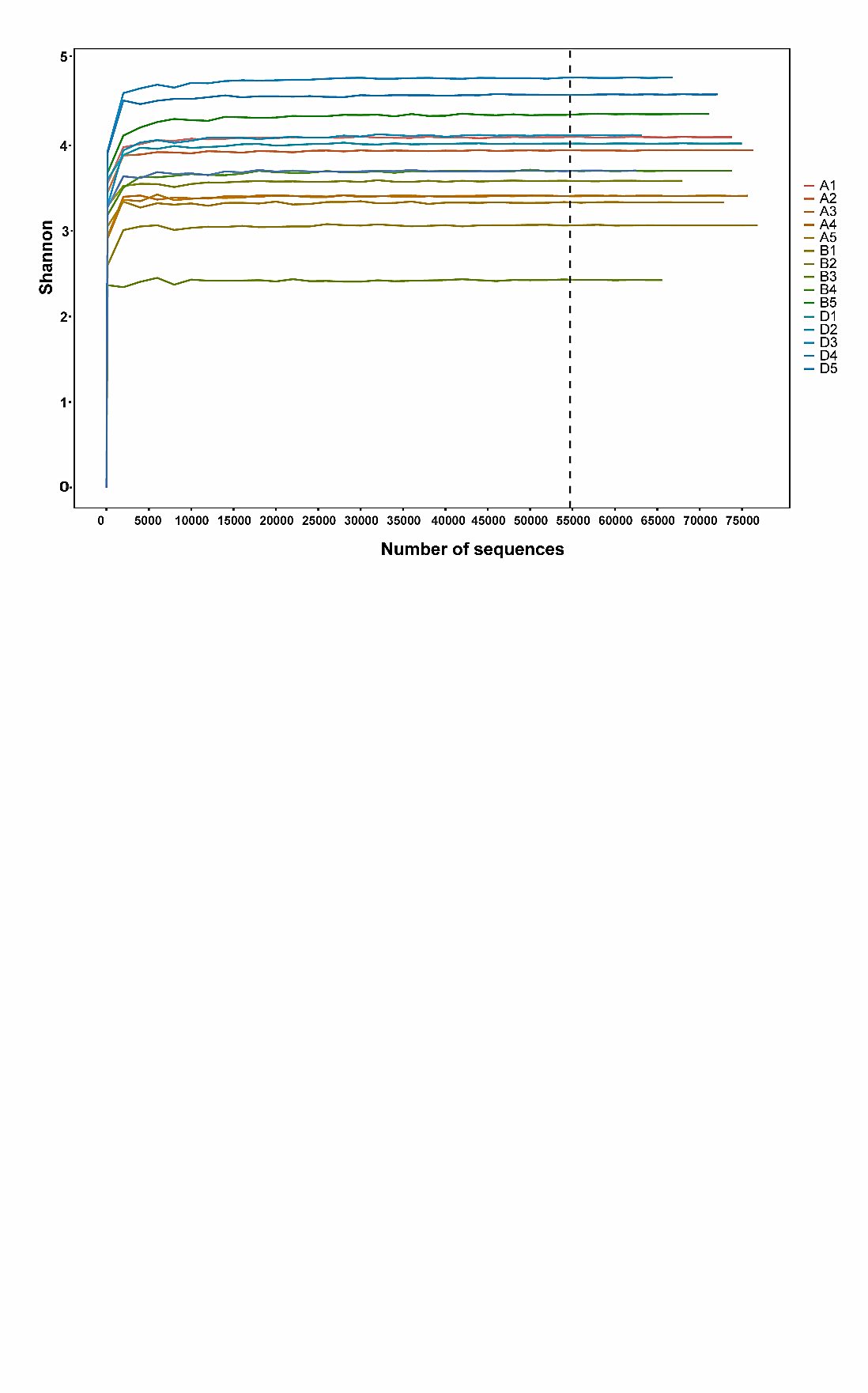


**Fig.S2 USM affected the GO and KEGG of liver transcriptome.** **(A)** Model vs Control KEGG enrichment analysis - up-regulation; **(B)** USM vs Model KEGG enrichment analysis – up-regulation; **(C)** Model vs Control KEGG enrichment analysis - down-regulation; **(D)** USM vs Model KEGG enrichment analysis - down-regulation.


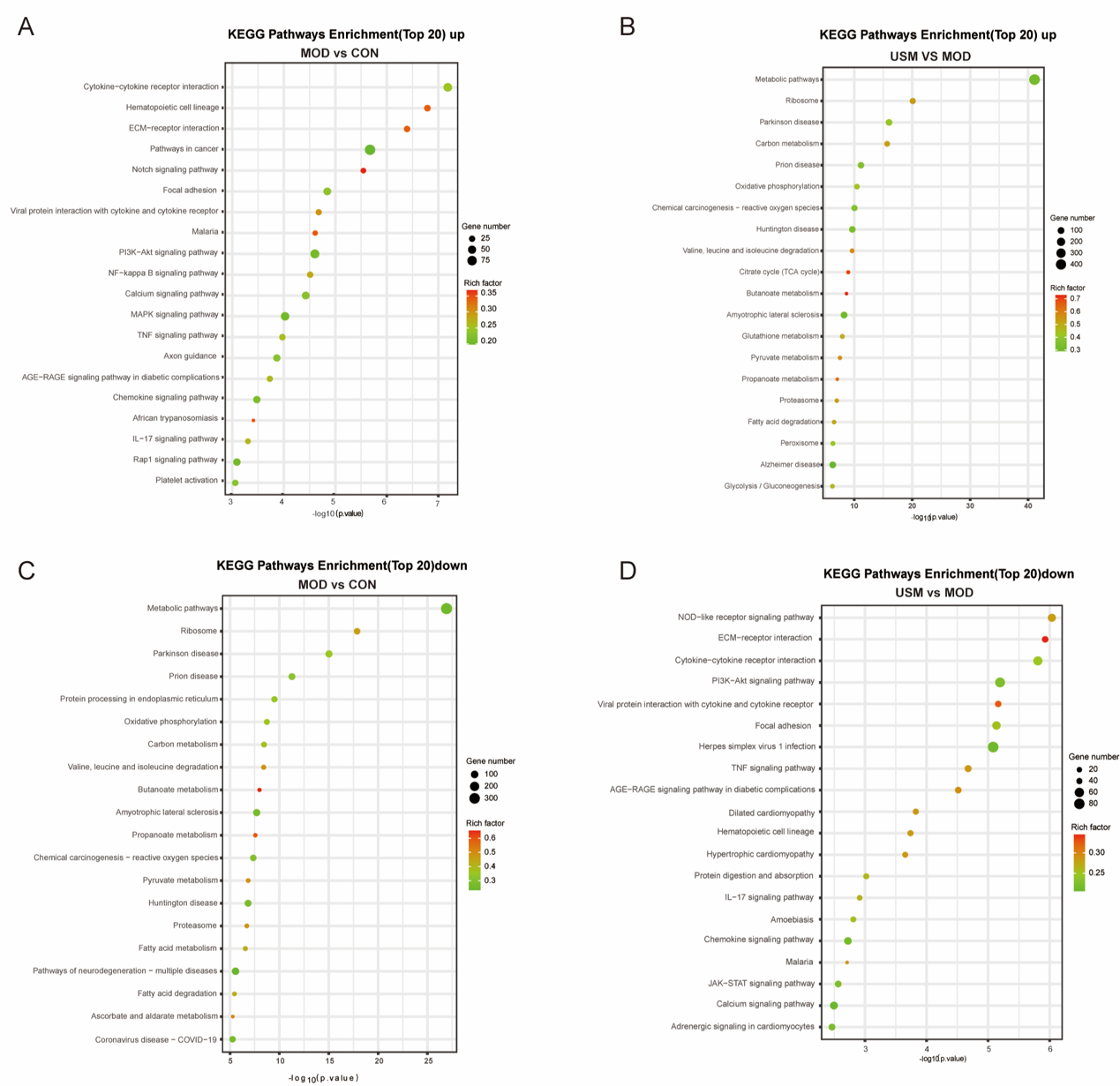

Supplement: Supplementary file 1 — Table S1: Primer sequences used for qRT‐PCR Analysis. Table S2: Raw data table of transcriptomics analysis. Figure S1: Shannon curves for mouse fecal samples. Figure S2: USM affected the GO and KEGG of liver transcriptome. (A) Model versus Control KEGG enrichment analysis—up‐regulation; (B) USM versus Model KEGG enrichment analysis—up‐regulation; (C) Model versus Control KEGG enrichment analysis—down‐regulation; (D) USM versus Model KEGG enrichment analysis—down‐regulation. [file FSN3-14-e71862-s001.docx]
